# Experience and personality modulate pupillary responses during real-time processing of within-language accent shifts

**DOI:** 10.1038/s41598-026-53089-z

**Published:** 2026-05-22

**Authors:** Adriana Hanulíková, Freya Gastmann, Sarah Schimke

**Affiliations:** 1 Institute for German as Foreign Language Philology, Heidelberg University, 69117 Heidelberg, Germany; 2 Institute for German Philology, LMU Munich, 80799 Munich, Germany; 3 Department of English Language & Culture, Center for Language and Cognition, University of Groningen, Groningen, 9712 EK The Netherlands

**Keywords:** Neuroscience, Psychology, Psychology

## Abstract

Speech perception shows substantial individual differences, but the mechanisms underlying this variability during real-time accent adaptation in natural conversation remain poorly understood. We used pupillometry to examine the temporal dynamics of processing effort while German listeners followed a semi-natural dialogue alternating between Standard German and an Alemannic regional variety of German. Growth curve analysis of the within-trial time course revealed switching costs and initial asymmetries, with greater effort for the regional variety and for switches from standard to regional accent. An analysis across the course of the dialogue further showed that the effect of accent driving these asymmetries diminished as listeners adapted to the regional variety. In contrast, switching costs remained stable across the dialogue, consistent with sustained attentional demands. Individual differences modulated these effects. A k-means cluster analysis yielded two clusters of listeners. The group characterized by greater exposure to Standard German, younger age, lower openness, and more favorable comprehensibility and pleasantness ratings for the standard speaker showed higher switching asymmetry, while overall switching costs did not differ reliably between profiles. These results are consistent with the interpretation that switching cost and switching asymmetry may reflect partly distinct underlying processes and that individual differences in language experience and personality selectively shape asymmetries during adaptation to within-first-language accent variation in real-time conversation.

## Introduction

Speech perception appears effortless, yet it involves complex cognitive processes that map highly variable acoustic signals to meaning^1^. This mapping process becomes especially challenging in conversations involving multiple speakers, because the perceptual system must not only adapt to each speaker’s accent and speaking style but also manage rapid alternation between speakers. Such speaker changes impose additional perceptual demands^2^, particularly in unfamiliar accents^3^. Variation in accents is ubiquitous, and all speakers vary depending on language background, age, social context, or social class^4^. Successful communication therefore depends on listeners’ ability to adapt to such variation, and there is ample evidence that experience and rich lexical knowledge support perceptual retuning of phonetic categories (e.g., ^5,^^6,^^7,^^8,^^9,^^10)^. However, the precise mechanisms that enable rapid adaptation in real time, and the factors that constrain this process during exposure to a quasi-naturalistic dialogue, remain unclear.

Recent studies underscore both the efficiency and the limits of rapid phonetic adaptation. Using an incremental exposure–test paradigm and Bayesian models, Tan and Jaeger^11^showed that rapid perceptual adaptation to shifted phonetic distributions can be best accounted for by distributional-learning theories, although adaptation often plateaus below the level predicted by ideal-observer models (e.g.,^12)^. Although their paradigm used controlled continua rather than natural accent variation, the underlying principle based on trial-by-trial updating of cue distributions provides a mechanistic framework that could apply to adaptation during extended conversational input. It suggests that listeners can update expectations quickly, but that there might be constraints on full recalibration of phonetic representations.

One major constraint is speaker and accent familiarity, which shapes real-time processing across multiple linguistic levels^3,13–16^. Accent familiarity effects have been interpreted in complementary frameworks (see^3)^, including auditory-streaming accounts of speaker change, talker normalisation, and exemplar-based models that attribute processing benefits to robustly represented accents. Using pupillometry, McLaughlin et al.^3^ found that switching between speakers and accents increases cognitive load compared to when the speaker and accent repeat, and that switching across accents is more effortful than switching within the same accent. Importantly, they observed an asymmetry in processing effort, with switches from a familiar first language (L1) accent to a less-familiar second language (L2) accent being more effortful than the reverse, and with processing of the L2 accent being overall more effortful than processing of the L1 accent. This pattern suggests that familiarity and experience are central to explaining switching effects, as listeners rely on stored representations to reduce control demands required to adapt to unfamiliar varieties. Similarly, numerous studies report increased processing effort for L2-accented speech that is attenuated by listener experience and extended exposure (e.g., ^3,^^16,^^17,^^18,^ but see ^19,20^, who report increased processing effort for L1-regional varieties compared to L2-accented speech).

McLaughlin et al.^3^ interpret their findings within two accounts of multi-talker adaptation. Within-accent switch costs are consistent with an auditory streaming account^21^, according to which listeners organize the acoustic input into perceptual streams based on acoustic continuity. Changes in speaker disrupt this continuity and require attentional reorientation to the new stream, resulting in processing cost that applies irrespective of accent familiarity. Across-accent switches, particularly from familiar L1 accents to less familiar L2 accents, cause additional demands that are not fully explained by the streaming framework alone. These are discussed in terms of an active-control mechanism involving talker normalization or phonetic recalibration, where listeners have to update their phonetic space to accommodate less familiar acoustic patterns. While any speaker change requires some degree of such accommodation, the demands depend on the distance between the listener’s phonetic representations and the incoming distribution, predicting greater costs for across-accent than within-accent switches. The observed switching asymmetry is also compatible with exemplar and episodic frameworks (e.g.,^22^^23)^, which predict processing benefits for familiar accents with robust representations in memory.

These findings raise a key question: Do switching asymmetries reflect stable processing differences between familiar and unfamiliar accents, or do they reflect temporary phonetic recalibration demands? This distinction has not been tested in longer, naturalistic conversational contexts, and the mechanisms and dynamics of perceptual adaptation within and across conversational dynamics are unclear. If asymmetries arise from stronger long-term representations for familiar varieties, they should persist throughout extended exposure to a conversation. Alternatively, if they reflect temporary recalibration demands - that is the time needed to establish a perceptual model of an unfamiliar phonetic distribution - they should diminish once listeners accumulate sufficient exposure. A possible approach to address this question is to monitor switching costs during extended listening to a quasi-naturalistic dialogue.

Most research on switching costs has focused on L1–L2 contrasts using isolated, highly controlled utterances. Such paradigms do not capture the demands of adaptation dynamics in ongoing conversation, and they rarely provide sufficient exposure to observe whether switching asymmetries change as listeners accumulate evidence about each speaker and their accent. In addition, little is known about how switching operates within L1 varieties, despite evidence that first-language varieties can differ substantially in intelligibility and processing demands (e.g.,^19,20,24)^. Some L1 varieties are less intelligible than L2 varieties when they diverge from a listener’s own variety and may impose greater processing demands than the standard. Whether switching between two L1 varieties yields costs similar to those observed in L1–L2 switching, and whether such costs are asymmetric, remains unknown. Most importantly, existing paradigms cannot determine whether switching effects reflect stable differences in architecture or dynamic adaptation. A quasi-naturalistic dialogue with hundreds of utterances and turn-takings would allow distinguishing between a stable architectural account and a dynamic adaptation explanation.

German provides an ideal testbed for addressing these questions. It is considered one of the most variable languages in Europe^25^, with German-speaking communities forming “a veritable gold mine for linguistic variation”^4^ (p. x). Across regions, listeners hear both Standard German and a continuum of regional accents and German dialects. These varieties differ in how often they are encountered, the extent to which they are acquired, and in the social meanings they carry (e.g.,^26^^27^^28)^. Of particular interest for the present study are subtle differences in phonetic detail, syllable reduction patterns, and intonation between Standard German and a regional Alemannic variety. We refer to this set of dynamic segmental and prosodic features in a person’s speech as accent^29^. Alemannic refers to a continuum of Upper-German dialects spoken in southwestern Germany and other neighboring German-speaking regions, with substantial internal regional variation^30,31^. Understanding how listeners navigate first-language accent switches in a language with a highly variable linguistic landscape is crucial for a comprehensive account of adaptation.

Importantly, adaptation processes may be shaped by individual differences. Traditionally, research in psycholinguistics has focused on commonalities across speakers, although individual differences in speech perception have been documented across multiple domains^32,33^. Such interindividual variability is not mere noise but a meaningful source for identifying the core mechanisms that underpin the acquisition and processing of language^34^. Individual differences may reflect relatively stable variation in both domain-general abilities such as working memory, prediction, and processing speed^35,36^, and language-specific experience, including vocabulary skills and sound-to-meaning mapping^19,20,32,37^. Such individual differences have important consequences for educational outcomes^38^ and social interactions^39^. Accordingly, understanding the sources of this variability in typical listeners is essential for identifying the factors that shape perception of accented speech and adaptation to switches between speakers.  However, no studies have specifically examined whether individual differences modulate switching effects, such as the magnitude or persistence of switching costs and asymmetry. Variation in language exposure (e.g., standard vs. dialect exposure) and properties of individuals’ social networks (e.g., the number and diversity of interaction partners) predict language comprehension across multiple linguistic levels, with benefits of heterogeneous input for processing both L2 accents^7,19^ and L1 accents^9,19,20,40^. Interacting with many different speakers can also improve phonological, semantic, and communicative skills^32,37,41^, an effect likely stemming from the need to adapt to different speakers. This is in line with usage-based frameworks, because social network properties relate to the variability of the linguistic input that individuals receive. However, it is unknown what type of variability in social networks facilitates adaptation in switching contexts.

In addition to social networks and exposure patterns, personality traits may also modulate speech processing and adaptation via affective and motivational responses to language variation^33,42^. We included this aspect as exploratory and were motivated by prior work suggesting that traits such as Openness, Extraversion, Agreeableness, and Neuroticism are associated with tolerance of ambiguity and responsiveness to unexpected or stereotyped forms^33,42^. Because accents carry social meaning, and listeners vary in their motivation and flexibility to accommodate different varieties^20^, personality provides a theoretically justified source of individual variation in adaptation during speaker switches.

Taken together, we lack an integrated understanding of (i) how switching costs and asymmetries operate within L1 varieties, (ii) how switching costs evolve during quasi-naturalistic dialogues tasks, and (iii) how individual differences in language experience, social networks, and personality traits relate to adaptation and switching costs in such contexts.

The present study examines how listeners adapt to a familiar Standard German accent and a less familiar regional Alemannic accent during a semi-natural dialogue, and whether switching cost and asymmetries emerge and evolve over time. We describe Standard German as familiar because participants are regularly exposed to this variety in everyday communication, higher education, and the media. We describe the Alemannic accent as less familiar because participants were recruited outside the specific speech community represented in the stimuli and were therefore expected to have little or no regular exposure to this particular variety. Importantly, Alemannic comprises a broader dialect continuum with substantial internal regional variation, such that familiarity with one subvariety does not necessarily imply familiarity with another.

A suitable method that assesses cognitive load as speech unfolds over time is pupillometry^43^. Pupil dilation is a well-established psychophysiological measure of cognitive and processing effort, with larger dilations reflecting greater demands on attentional and cognitive resource ^43,44^. We use the term cognitive effort to refer to a set of cognitive demands imposed by a given task, which can arise from multiple processes including attentional reorientation and phonetic recalibration. In the current study, we use pupil dilation as an index of overall processing effort of accented speech and examine the contributions of distinct mechanisms through their temporal dynamics and association with individual listener profiles. Importantly, pupil dilation does not allow direct identification of specific underlying mechanisms but instead reflects their combined influence. To investigate these questions, we tracked real-time processing effort as L1 German listeners listened to a quasi-naturalistic conversation varying in turn-taking, utterance length, accent, and (morpho)syntax, which represented an ecologically more valid context over the highly controlled single-utterance paradigms used in previous studies. To keep participants engaged throughout the dialogue, participants performed a grammatical acceptability task on each utterance (balanced across accents and switch conditions). This task addressed another research question not included in this paper. In addition, we assessed lexical skills, social network size, exposure to German varieties as well as other languages, and personality traits, because all of these factors have been shown to modulate speech processing. We employed growth-curve analysis (GCA) to model the group-level temporal trajectory of switching costs, a trial-level analysis to assess temporal dynamics throughout the dialogue, and k-means clustering to identify distinct listener profiles. This combination of analytic approaches allows us to address the following main questions: Do switching costs and asymmetries occur within L1 German varieties? Do they show distinct temporal dynamics? And do listeners cluster into profiles reflecting different exposure histories and adaptation strategies?

We predicted greater processing effort (larger pupil dilation) for the Alemannic speaker than the Standard German speaker, and larger costs when switching from Standard to Alemannic than vice versa, similar to previously found L1–L2 asymmetries attributed to accent familiarity. Critically, if switching asymmetries reflect temporary perceptual recalibration rather than stable representational differences, they should diminish across the dialogue as listeners adjust their priors and adapt to the speaker’s phonetic distribution. Under a distributional-learning account, asymmetry should decline as cue-distribution uncertainty decreases with exposure. In contrast, general switching costs reflecting attentional demands should remain stable. Finally, we expect listener profiles to reflect distinct strategies for processing accent variation during conversation.

## Results

### Switching costs and asymmetries

**Fig. 1:**
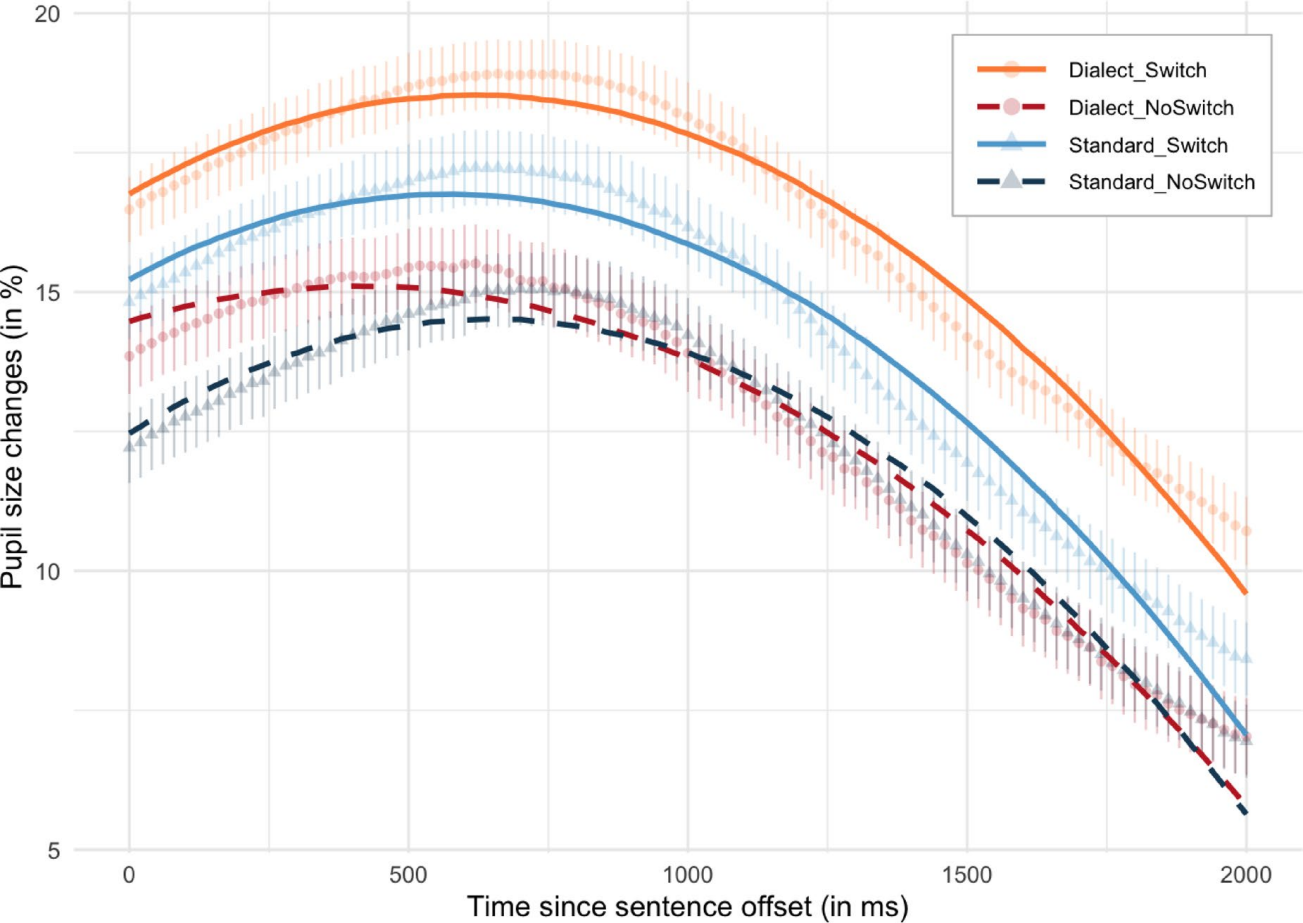
The interaction between Switch and Speaker is shown with GCA model fits (solid and dashed lines) and raw data means (points and triangles). Range displays 95% confidence intervals.

Figure 1 shows the interaction between Switch condition and Speaker across the full 271-trial dialogue, plotted separately for Standard German and the Alemannic variety. Visual inspection reveals clear switching costs, with switch trials eliciting larger pupil dilations than non-switch trials across both accents. The largest dilations occur for standard-to-dialect switches, whereas dialect-to-standard switches produce smaller responses, suggesting asymmetric processing demands favoring the familiar standard variety.

The growth curve analysis (0–2000 ms after utterance offset) confirmed these visual patterns. There were main effects of Switch, Speaker, and first- and second-order polynomial terms (see Table 1). Switch trials elicited greater pupil dilations than non-switch trials (β = − 1.33, *SE* = 0.27, *t* = − 4.92, *p* <.001), and dialect trials elicited greater dilations than standard trials (β = 0.72, *SE* = 0.27, *t* = 2.70, *p* =.007). A main effect of Trial indicated an overall decline in pupil size across the experiment (β = − 1.71, *SE* = 0.32, *t* = − 5.40, *p* <.001), likely reflecting general adaptation or task-related factors. Together, these findings demonstrate that speaker alternation and regional accent independently increase processing effort, extending switching-cost and accent-familiarity effects previously reported for L1–L2 contrasts^3^ to variation within L1 varieties.

Several interactions further modulated the pupillary time course. A significant two-way interaction emerged between Switch × quadratic polynomials (β = 0.67, *SE* = 0.16, *t* = 4.32, *p* <.001), indicating steeper acceleration and more convex rise-and-fall trajectories for switch relative to non-switch trials. Two-way interactions between Speaker × linear polynomials (β = − 0.65, *SE* = 0.16, *t* = − 4.19, *p* <.001) and Speaker × quadratic polynomials (β = 0.92, *SE* = 0.16, *t* = 5.90, *p* <.001) further revealed that dialect trials show a more rapid decline in pupil size over time, whereas standard trials show greater curvature in their dilation–constriction pattern.

Crucially, significant three-way interactions between Switch × Speaker × linear polynomials (β = − 2.14, *SE* = 0.16, *t* = − 13.73, *p* <.001) and Switch × Speaker × quadratic polynomials (β = 0.83, *SE* = 0.16, *t* = 5.36, *p* <.001) indicate that the effect of switching on pupil size changes over time and depends strongly on the speaker. Specifically, the linear slope of the pupil response is less steep for switch trials when listening to the dialect speaker than to the standard speaker. Likewise, the curvature of the pupillary response is more convex for standard-to-dialect switches than for dialect-to-standard switches. This pattern reflects greater processing demands when switching from the familiar standard to the less familiar regional variety, consistent with an asymmetry driven by accent familiarity and phonetic recalibration. Growth curve model output is displayed in Table 1 and Fig. 1.

**Table 1:** Model output for growth curve analysis.

	Estimate	*SE*	*t*	*p*
(Intercept)	13.88	0.85	16.28	0.000
**poly1**	**−22.43**	**0.16**	**−144.22**	**0.000**
**poly2**	**−13.79**	**0.16**	**−88.63**	**0.000**
**switch1**	**−1.33**	**0.27**	**−4.92**	**0.000**
**speaker1**	**0.72**	**0.27**	**2.70**	**0.007**
**scale(trial)**	**−1.71**	**0.32**	**−5.40**	**0.000**
poly1:switch1	−0.09	0.16	−0.59	0.533
**poly2:switch1**	**0.67**	**0.16**	**4.32**	**0.000**
**poly1:speaker1**	**−0.65**	**0.16**	**−4.19**	**0.000**
**poly2:speaker1**	**0.92**	**0.16**	**5.90**	**0.000**
switch1:speaker1	−0.30	0.26	−1.16	0.247
**poly1:switch1:speaker1**	**−2.14**	**0.16**	**−13.73**	**0.000**
**poly2:switch1:speaker1**	**0.83**	**0.16**	**5.36**	**0.000**

Note that significant effects are printed in bold.

### Temporal dynamics across the dialogue

**Fig. 2:**
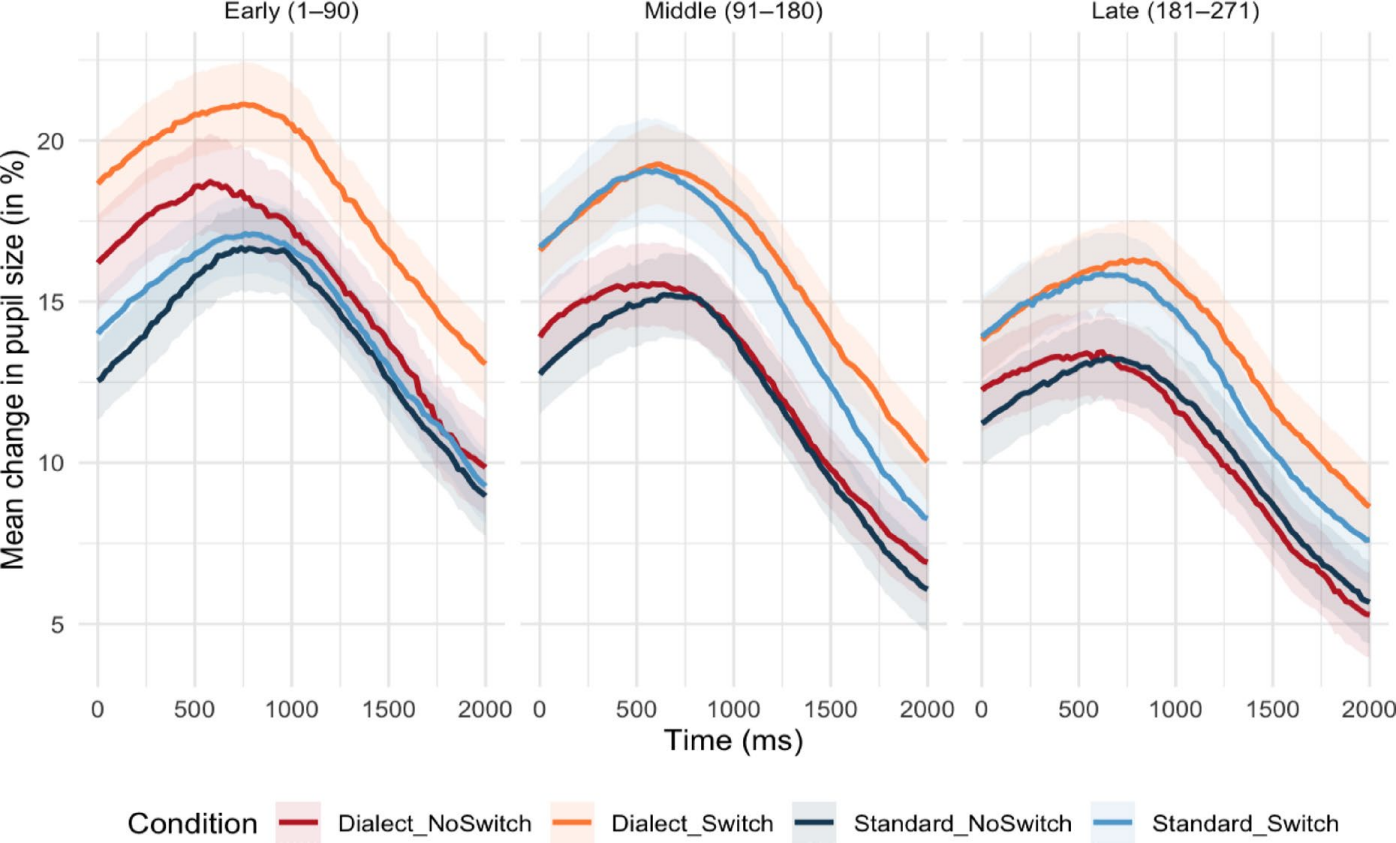
Average change in pupil sizes over time. Note that the descriptive data was plotted for three time windows for better visual interpretation only. Shaded areas represent 95% confidence intervals.

Figure 2 illustrates that switching asymmetry appears robust in Block 1 but is reduced in Block 2 and Block 3. In contrast, general switching costs remained stable across all blocks. This visual pattern is consistent with the interpretation that attentional reorientation persists throughout the dialogue, whereas phonetic recalibration demands diminish as listeners adapt to the dialect. To statistically test whether switching costs and asymmetries change over the course of the dialogue, we performed linear mixed-effects analysis on pupil size by Switch x Speaker x Trial (see Table 2). In line with the GCA, the results revealed main effects of all three predictors, with switch trials eliciting greater pupil dilations than non-switch trials (ß = 1.26, *SE* = 0.27, *t* = 4.62, *p* <.001), dialect trials inducing greater dilations than standard trials (ß = 0.76, *SE* = 0.27, *t* = 2.82, *p* =.005), and an overall decline in pupil dilations over the course of the experiment (ß = − 1.74, *SE* = 0.31, *t* = − 5.56, *p* <.001), likely reflecting pupil fatigue (see ^45^). Furthermore, the analysis revealed a significant two-way interaction of Speaker x Trial (ß = − 0.64, *SE* = 0.25, *t* = − 2.51, *p* =.013), indicating that, as illustrated in Fig. 2, the effect of speaker diminished over the course of the experiment, with the difference in pupil dilations in response to the dialect and standard speaker gradually decreasing over time. In contrast, none of the interactions with Switch was significant. Because the model averages across the pupil time course within each trial, it disregards the within-trial temporal dynamics. It therefore likely has less sensitivity to detect asymmetry effects compared to the GCA, which captures asymmetry through the Switch × Speaker × Polynomial interaction.

**Table 2:** Model output for linear mixed effects analysis of pupil size by Switch x Speaker x Trial.

	Estimate	*SE*	*t*	*p*
(Intercept)	13.93	0.86	16.23	0.000
**switch1**	**1.26**	**0.27**	**4.63**	**0.000**
**speaker1**	**0.76**	**0.27**	**2.82**	**0.005**
**scale(trial)**	**−1.74**	**0.31**	**−5.56**	**0.000**
switch1:speaker1	0.23	0.25	0.92	0.357
switch1:scale(trial)	0.27	0.26	1.02	0.308
**speaker1:scale(trial)**	**−0.64**	**0.25**	**−2.51**	**0.013**
switch1:speaker1:scale(trial)	−0.15	0.26	−0.60	0.548

Note that significant effects are printed in bold.

### Listener profiles

**Fig. 3:**
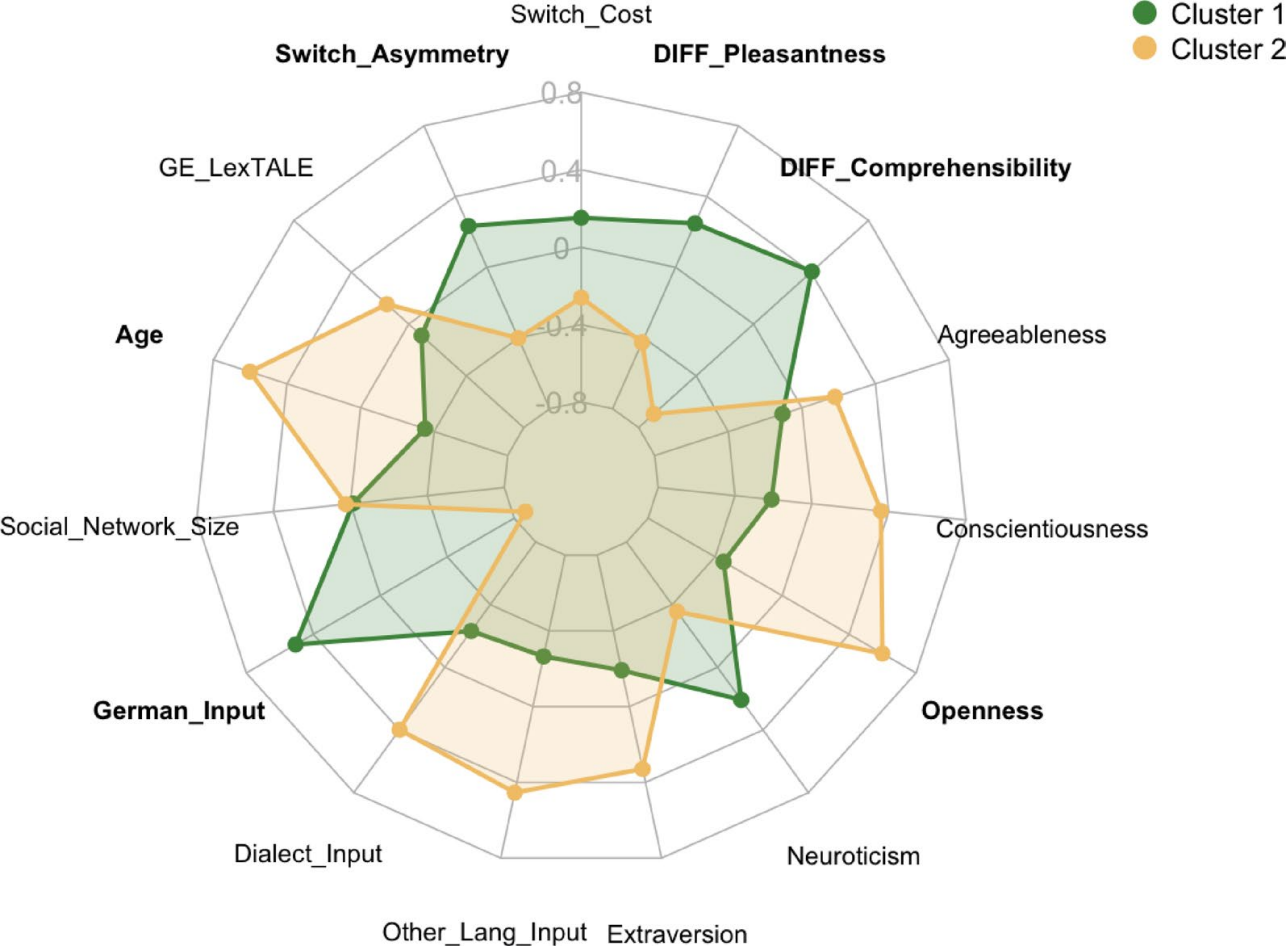
Cluster comparison chart. Significant variables after Holm-Bonferroni correction are printed in bold.

To examine whether listeners cluster into profiles reflecting different exposure histories and adaptation strategies, we conducted k-means cluster analysis. The final analysis yielded two clusters of different sizes (Cluster 1: *n* = 48; Cluster 2: *n* = 28) that differed robustly in switching asymmetry, age, Standard German exposure, openness, and in the perception of comprehensibility and pleasantness of the two speakers. Accordingly, cluster 1 consists of a slightly younger population with comparatively more Standard German input. In terms of personality, participants in cluster 1 appear to be less open. Furthermore, cluster 1 exhibits higher switch asymmetry, that is greater difficulty switching from standard to dialect than vice versa. In addition, participants in cluster 1 displayed higher comprehensibility and pleasantness difference scores, indicating that on average, they showed a stronger preference for the standard over the regional speaker in their ratings of comprehensibility and pleasantness than participants in cluster 2. Cluster 2, on the other hand, is characterized by a slightly higher average age, less standard German input, and a comparatively more open personality. Additionally, cluster 2 exhibits less switch asymmetry, meaning that they have less difficulty with switching into dialect than participants in cluster 1, and they had lower comprehensibility and pleasantness difference scores, indicating a weaker preference for the standard speaker. Table 3 shows full statistics; Fig. 3 visualizes the cluster separation.

**Table 3:** Model output for k-means cluster analysis.

	Mean (Cluster 1)	Mean (Cluster 2)	*t*	*p*	*p* (Holm)	*d*
Switch_Cost	3.36	1.98	1.75	0.085	0.339	0.41
**Switch_Asymmetry**	**3.13**	**0.15**	**2.97**	**0.004**	**0.045**	**0.66**
GE_LexTALE	85.63	87.32	−0.96	0.341	0.682	−0.24
**Age**	**21.75**	**25.27**	**−3.90**	**0.000**	**0.005**	**−1.06**
Social_Network	9.77	10.11	−0.16	0.876	0.876	−0.04
**German_Input**	**81.21**	**49.88**	**7.20**	**0.000**	**0.000**	**1.81**
Dialect_Input	3.65	13.04	−2.32	0.027	0.159	−0.65
Other_Lang_Input	11.48	24.66	−2.83	0.007	0.065	−0.76
Extraversion	2.57	2.98	−2.22	0.030	0.159	−0.53
Neuroticism	3.04	2.61	2.52	0.014	0.116	0.58
**Openness**	**3.02**	**3.73**	**−5.12**	**0.000**	**0.000**	**−1.05**
Conscientiousness	2.81	3.21	−2.49	0.016	0.116	−0.58
Agreeableness	2.81	3.00	−1.30	0.197	0.590	−0.28
**DIFF_Comprehensibility**	**2.42**	**0.75**	**6.09**	**0.000**	**0.000**	**1.28**
**DIFF_Pleasantness**	**2.29**	**0.71**	**2.99**	**0.004**	**0.045**	**0.70**

Note that significant effects after Holm-Bonferroni correction are printed in bold.

## Discussion

The present study examined how listeners adapt to accent variation while listening to a quasi-natural dialogue alternating between Standard German, a highly familiar accent, and a regional Alemannic variety, a less familiar accent. Using pupil dilation as an index of processing effort, we addressed two central questions: Do switching costs reflect stable processing differences or transient adaptation demands? And how do individual differences in language experience, social networks, and personality shape adaptation processes?

We found switching costs and switching asymmetry within L1-regional accents, extending L1-L2 switching research^3,15,17^ to first-language varieties. The Alemannic accent and switch trials elicited greater processing effort than Standard German and non-switch trials. However, the asymmetry was mainly concentrated in switch trials: switching from standard to dialect required substantially greater effort than switching from dialect to standard, whereas the difference between accents in non-switch trials was more modest. This pattern indicates that asymmetric processing demands arise primarily during transitions into the less familiar accent, in line with active recalibration rather than global processing difficulty alone. A global processing difficulty account would predict higher effort for the regional accent persisting across both switch and non-switch trials during the dialogue, and hence, symmetric effects. These results further suggest that accent familiarity, rather than L1/L2 status per se, drives asymmetric adaptation demands.

A central question is whether switching asymmetries reflect stable differences in representational strength linked to long-term accent familiarity or temporary phonetic recalibration demands that diminish as exposure accumulates. The adaptation analysis provides evidence for the latter. Visual inspection of the fitted trajectories shows that the asymmetry (greater cost for standard-to-dialect switches than vice versa) was robust in the early part of the dialogue but diminished after approximately one third of trials (see Fig. 2). However, this asymmetry was confirmed only in the more sensitive GCA through the Switch × Speaker × Polynomial interaction, which captures differences in the shape of the pupil response over time rather than its mean. The significant Speaker × Trial interaction in the linear mixed-effects analysis provides complementary support, showing that the overall effect of accent diminished over the course of the dialogue, while switching costs did not show a comparable change. Because pupillometry indexes a mix of cognitive effort, attention, and control allocation, the results cannot directly isolate effects of belief-updating. Nevertheless, the time course of the switch asymmetry is descriptively and qualitatively consistent with distributional-learning and ideal-adapter accounts of rapid spoken-language adaptation^11,12,46^, which propose that listeners continuously update expectations about a speaker’s phonetic cue distribution as they accumulate evidence. Early in the dialogue, the less familiar Alemannic accent generates high uncertainty and therefore larger belief updates, producing increased switching costs. As exposure accumulates, listeners refine their estimates of the speaker’s cue distributions and adjust their priors accordingly, reducing switching asymmetry.

In contrast, general switching costs showed no comparable temporal modulation across the dialogue, consistent with auditory-streaming accounts, according to which an attentional-reorientation process tied to speaker change is required even for more familiar accents^ 3^. Thus, the temporal dissociation between relatively stable switching cost and diminishing cost asymmetry supports the idea that these effects arise from distinct underlying mechanisms. One of the possible mechanisms is tied to attentional reorientation and one to distributional recalibration.

An inspection of the temporal trajectory analysis revealed descriptively elevated processing costs for switching into Standard German in later trials. Although this pattern was not supported by the three-way interaction in the linear mixed-effects analysis, it appeared selectively in one condition and is therefore unlikely to reflect fatigue. However, this observation should be interpreted with caution. One possible account is that, once listeners have reduced their uncertainty about the Alemannic speaker’s cue distributions, subsequent input from the standard speaker requires recalibration toward a different, less recently updated distribution. Under distributional-learning and ideal-adapter accounts^11,12^, belief updating is strongest for distributions that start with high uncertainty. Because the Alemannic accent begins with higher uncertainty (and likely even more so for standard-dominant listeners), Alemannic trials carry higher informational weight early on. As listeners stabilize their estimates of the regional accent, switching back to Standard German requires recalibration of expectations. This process occurs not because the standard is less familiar overall, but because its distribution has been less recently updated. Thus, the increased standard-switch effort in later trials might reflect dynamic adjustment of expectations as the system accommodates different speakers in an extended dialogue.

An alternative contributing factor to the early asymmetry could be grammatical expectations related to the two varieties^13,40^. Because participants performed acceptability judgments (in contrast,^3^ used sentence repetition), and because Standard German is associated with prescriptive norms, listeners may initially have attributed the regional accent to a higher likelihood of non-standard syntax and may have been less surprised to encounter variation. Although grammatical structures were fully crossed with speaker, such expectations may nevertheless have influenced early responses. However, several aspects of the results argue against this factor as the primary explanation. First, listeners are known to adapt rapidly to syntactic variation, showing reduced rather than increased processing effort over time^13^. Second, grammar-based monitoring should affect processing regardless of whether the speaker changes. In contrast, we observed higher processing effort in switch trials compared to non-switch trials, whereas recalibration demands arise specifically during transitions. This pattern of results is more parsimoniously explained by dynamic phonetic recalibration: initial asymmetry (less familiar phonetic distribution), rapid adaptation (belief updating), and later standard-switch increase (recalibration to less-recently updated distributions).

Individual differences further clarify how listeners process accent variation in conversation. The k-means cluster analysis revealed two coherent listener profiles that differed systematically in switching asymmetry, language exposure, personality traits, and subjective accent evaluations. Importantly, these profiles did not differ reliably in overall switching costs, suggesting that they reflect distinct adaptation strategies rather than general processing capacity differences.

The group showing significantly larger switching asymmetry (63% of the sample) was younger, more standard-dominant, less open, and exhibited larger perceived differences in pleasantness and comprehensibility between speakers. This profile is consistent with a pattern that may reflect an adaptation strategy optimized for standard-dominant contexts. It indicates robust standard representations that facilitate baseline processing of the standard variety, but simultaneously, their limited Alemannic exposure results in large recalibration demands when switching into less familiar regional varieties. The combination of lower openness and stronger preference for standard speakers suggests both reduced exposure to regional variation and reduced motivation or flexibility to accommodate less familiar regional forms. These factors may reinforce each other in shaping adaptation patterns.

In contrast, the low-asymmetry group (37% of sample) was slightly older, more open, descriptively exposed to more diverse input (more other languages and dialects), and showed weaker preferences between the two accents. This profile appears optimized for linguistically diverse environments, with more balanced representations across varieties enabling flexible switching without a strong directional cost. Higher openness is consistent with accounts linking this trait to cognitive flexibility, reduced aversion to ambiguity, and greater willingness to accommodate variability^33,42^. Although switching cost showed only a weak and non-significant tendency toward reduction in the lower asymmetry group, the descriptive pattern suggests that the two components of processing cost are not fully independent. Switching asymmetry appears more sensitive to experience-driven recalibration, whereas switching cost reflects a relatively stable attentional component that may require larger differences in experience between groups or longer exposure to show group-level effects.

Importantly, both profiles appear consistent with adaptation to different communicative environments. The standard-oriented profile is characterized by stronger alignment with standard-dominant contexts, while the language-flexible profile is more strongly aligned with linguistically heterogeneous contexts (dialectal regions, multilingual environments). These individual differences align with usage-based frameworks^22,23^, which suggest that linguistic representations are shaped by accumulated experience and that frequent exposure to variable input enhances flexible phonetic-lexical mappings^19^. Following usage-based models, “linguistic units are gradient categories that have no fixed properties but rather are formed on the basis of experienced tokens,” and experience “thus has an ongoing effect on mental representation”^23^ (p. 830). In line with this idea, increased exposure to variable input, including regional accents, may support more flexible acoustic–lexical mapping during accent switches. However, this does not imply that phonetic representations are easily overwritten. Rather, they are shaped by the cumulative history of experience and are therefore expected to be relatively stable, particularly in adult L1 users. The effects observed here are more plausibly explained in terms of temporary adjustments in mapping operations or perceptual weighting, rather than restructuring of phonetic representations. The emergence of two listener profiles suggests that adaptation to accent variation during conversation is not uniform but shaped by individual histories and socio-cognitive dispositions. This is also consistent with Communication Accommodation Theory, which proposes that individuals differ in their flexibility and motivation to accommodate linguistic variation. Our findings also parallel recent work by Kutlu et al.^47^, who identified gradient versus categorical speech perception profiles in children based on linguistic diversity in their social networks. Together, these studies suggest that speech perception strategies are not uniform but tuned to individual linguistic ecologies, which have important implications for understanding both typical variation and atypical processing.

Together, the results demonstrate that adaptation to regional accent variation in a quasi-naturalistic dialogue operates through two partially independent mechanisms with distinct temporal dynamics. General switching costs are consistent with sustained attentional reorientation tied to speaker changes, a process required regardless of accent familiarity and relatively insensitive to short-term exposure effects (at least for this sample). These costs remained stable throughout the dialogue. Switching asymmetries, in contrast, are consistent with transient phonetic recalibration triggered specifically when switching into an unfamiliar accent. This asymmetry was characterized by greater costs when switching into the less familiar accent than vice versa. This effect showed a reduction after the first third of the listening task, rather than persisting as would be predicted by a global processing difficulty account. The dissociation between persistent general costs and transient asymmetries is consistent with the interpretation that accent-familiarity effects reflect adaptation rate rather than permanent architectural differences.

These patterns were modulated by individual differences. Language exposure shaped asymmetry magnitude, with standard-dominant listeners showing larger initial recalibration demands, but did not reliably affect general switching costs. Personality traits like openness predicted accommodation flexibility. The emergence of two distinct listener profiles suggests that adaptation strategies are shaped by individual linguistic ecologies, with each profile being consistent with alignment to different communicative environments.

Several limitations warrant consideration. First, our sample comprised primarily young university students with limited dialectal diversity, potentially restricting generalizability to broader populations. The Alemannic variety was unfamiliar to most participants, meaning that they had no or very limited experience with the specific Freiburg-region variety, preventing bidirectional familiarity comparisons. Second, a critical question for intervention research is whether listener profiles are stable traits or whether listeners can be trained to shift strategies. The temporal dynamics findings suggest potential for malleability, indicating that even standard-oriented listeners can adapt with sufficient exposure. However, individual differences in adaptation rate (how quickly asymmetry disappears) were not directly tested here. We leave this question to future research. Finally, pupillometry provides an indirect index of cognitive effort that reflects overlapping influences of attention, uncertainty, and control demands, which constrains the ability to attribute effects to specific computational mechanisms. In future research, we plan to examine switching costs across multiple regional varieties with varied familiarity levels and more diverse participant populations, including second-language listeners, who typically struggle most when exposed to regional varieties. Investigating second-language learners will illuminate how individual differences in switching costs and asymmetries relate to proficiency and exposure history.

## Conclusion

This study shows that switching costs and switching asymmetries extend beyond L1-L2 contrasts to processing of regional varieties within a first language. Critically, adaptation to accent switches in a quasi-naturalistic dialogue is shaped by two distinct but related mechanisms: a sustained attentional cost of speaker change, as well as a transient recalibration cost that is concentrated in switching into the unfamiliar accent, depends on accent familiarity, and diminishes with accumulated exposure during conversation. Individual variation in language experience, evaluative judgments, age, and personality modulate the switching asymmetry, giving rise to qualitatively distinct listener profiles. These findings advance our understanding of real-time adaptation to sociolinguistic variation in a dialogue and highlight how experience and socio-cognitive dispositions jointly shape the dynamics of spoken-language processing.

### Method

The study protocol was approved by the research ethics committee of the DGFS (Deutsche Gesellschaft für Sprachwissenschaft „German Linguistics Society”, Number: 2024–20) and performed in accordance with the Declaration of Helsinki.

### Participants

Ninety-six L1 German students of two universities in Southern Germany (Heidelberg and Munich) participated in this study. All participants gave informed consent and either received study credits or a small monetary compensation for their participation.

All participants completed the German version of the LexTALE task, the pupillometry experiment, a debriefing questionnaire, and a background questionnaire. The LexTALE task^48 ^assesses receptive vocabulary knowledge as a proxy for general language proficiency in Standard German. The debriefing questionnaire was a paper-and-pencil form assessing participants’ perceptions of the experimental task and their social evaluation of the two speakers. It included two questions that elicited ratings of the comprehensibility and pleasantness of the two speakers on a scale from 1 to 10. Based on this, a difference score was compiled, with higher values indicating a relatively higher degree of comprehensibility or pleasantness for the speaker with the standard compared to the one with the regional accent. The background questionnaire was adapted from a questionnaire used in Levy et al.^19^ , which assessed basic sociodemographic and language background data, and was implemented in a digital format using the software *SoSciSurvey*^49^. Participants estimated the percentage of Standard German input, input in dialects, and input in other languages that they receive in a typical week. In addition, participants indicated the size of their social network, operationalized as the number of people they interact with in a typical week. Finally, the questionnaire included items assessing the “Big Five” personality traits^50^. Note that the original 5-point Likert scale was reduced to 4 points to achieve clearer differentiation between agreement and disagreement (see^51^, for a similar procedure).

All participants reported normal hearing and normal or corrected-to-normal vision. Eleven participants had to be excluded due to either missing data from the background tasks (4) or the pupillometry experiment (7). An additional nine participants were excluded due to a high amount of data loss after pupillometry data preprocessing (see analysis section below). Of the remaining 76 participants (71 women, 5 men), 56 grew up with (standard and/or dialectal) German as their sole L1, while 20 participants grew up bilingually with German and another first language. Thirty-one participants indicated regular use of a dialect, of which 17 indicated “Bavarian”, and 14 mentioned various dialects (with only one of them labeling their dialect as “Alemannic”; however, that speaker came from a different region than the present study’s dialect speaker). Most participants reported knowledge of further foreign languages, in particular English. Table 4 summarizes further information about the sample.

**Table 4:** Participant characteristics (*n* = 76).

	*M*	*SD*	Range
Age (years)	23.1	3.7	18–36
German proficiency (GE LexTALE)	86.3	7.0	65.0–97.5
Standard German input (in %)	69.7	22.8	15–100
Dialectal input (in %)	7.1	14.9	0–70
Other language input (in %)	16.3	18.3	0–80
Social network size^a^	9.9	8.9	3–50
Big Five personality traits^b^			
Extraversion	2.7	0.8	1.0–4.0
Neuroticism	2.9	0.8	1.5–4.0
Openness	3.3	0.8	1.5–4.0
Conscientiousness	3.0	0.7	1.5–4.0
Agreeableness	2.9	0.7	1.0–4.0
Difference in comprehensibility (DIFF Comprehensibility)^c^	1.8	1.5	0.0–5.0
Difference in pleasantness (DIFF Pleasantness)^d^	1.7	2.3	−6.0–7.0

^a^ Average number of conversational partners per week.

^b^ Personality traits on a scale from 1 to 4, with higher values indicating greater applicability of the personality trait.

^c^ Difference score concerning comprehensibility ratings. Comprehensibility was measured on a scale from 1–10. The difference score was determined by subtracting the value for the regional speaker from the value for the standard speaker (e.g., higher values reflect higher standard orientation).

^d^ Difference score concerning pleasantness ratings. Pleasantness was measured on a scale from 1–10. The difference score was determined by subtracting the value for the regional speaker from the value for the standard speaker (e.g., higher values reflect higher standard orientation).

### Materials

The auditory materials for the pupillometry study consisted of a semi-natural dialogue, originally created for a grammatical acceptability judgement study^40^. The dialogue transcript stemmed from the Research and Teaching Corpus of Spoken German (FOLK^52)^ and was substantially adapted to translate colloquial features into forms closer to Standard German. It contained 278 utterances of varying length produced by two speakers discussing everyday topics. The dialogue was recorded by one speaker using Standard German accent (standard speaker), while the other exhibited phonetic and phonological features of an Alemannic variety spoken in the Freiburg region in Germany (dialect speaker). The Alemannic language area is a continuum of Upper-German dialects spoken in southwestern Germany (and other German-speaking regions, such as Switzerland). Alemannic does not exhibit uniform phonological or morphological features that would allow for a consistent classification of Alemannic as a whole or for distinguishing it from neighboring dialects^31^. The phonological and lexical aspects have been well documented, whereas the syntactic aspects have been studied to a lesser extent^40^. All lexical items in the dialogue were Standard German, ensuring intelligibility even for participants unfamiliar with Alemannic dialects. In the original study, the standard speaker’s accent was predominantly classified as Standard German, with some listeners perceiving a slight Swabian or Baden regional coloring in the pronunciation. The dialect speaker’s accent was almost unanimously categorized as Alemannic, Swabian, or Baden, with only one exception (see^40)^. Both speakers were rated for intelligibility on a scale from 1 (highly intelligible) to 7 (not intelligible), with the standard speaker being descriptively more intelligible (*M* = 1.44) than the dialect speaker (*M* = 2.10).

The first seven utterances of the dialogue served as practice trials to familiarize participants with the speakers and the task. These utterances were excluded from analysis. Of the remaining 271 utterances, 160 were carefully controlled for morphosyntactic features. These utterances instantiated different (morpho)syntactic contrasts between Standard German and Alemannic, where the dialectal variants deviate from Standard German. The original study varied and examined the salience of regional variants as a function of the accent. Including these dialectal variants made sure that speakers could give both “yes” and “no” answers in the acceptability judgement task. Importantly, each of these utterances was recorded by both speakers in both the standard and the dialect morphosyntactic variants, and the two versions of the utterances by each speaker were distributed equally across experimental lists. This means that speaker accent was not systematically associated with regional variants. The remaining 111 utterances of the dialogue did not contain any dialectal (morpho)syntactic features and were included in the original study to maintain a coherent flow of conversation.

Throughout the dialogue, 130 utterances followed an utterance from the same speaker (no-switch trials), and 141 utterances followed a change in speaker (switch trials). There were 65 no-switch trials and 70 switch trials for the standard speaker, and 65 no-switch trials and 71 switch trials for the dialect speaker. An excerpt of the dialogue is shown in Table 5 as an illustration.

**Table 5:** Excerpt of the dialogue to illustrate experimental conditions.

Speaker Accent	Switch	Utterance
Standard	no switch	Hast du nicht gut geschlafen? ‘Did you not sleep well?’
Dialect	switch	Ich war heute Nacht sehr überrascht, wo es gewittert hat. ‘I was very surprised last night when there was a thunderstorm’
Dialect	no switch	Als es so stark geblitzt hat, habe ich mich sehr gefürchtet. ‘When there were lightning flashes, I was very scared’
Dialect	no switch	Der Wind, wo um das Haus geblasen hat, war sehr furchteinflößend. ‘The wind that blew around the house was very frightening’
Standard	switch	Irgendwann habe ich mitbekommen, dass da etwas war. ‘At some point I noticed that something had happened’
Standard	no switch	Aber da habe ich wahrscheinlich geschlafen gehabt. ‘But I was probably asleep at the time’
Dialect	switch	Das war heute Morgen so um sechs Uhr, als es richtig los ging. “It was around six in the morning, when it really started’

To make the listening task more engaging, more natural, and less effortful, participants looked at photographs of two male faces during the experiment, representing the two speakers. Whenever there was a switch between speakers in the dialogue, the picture on the screen switched as well. Note that this switch happened at the beginning of each trial and prior to the baseline measurement, such that it could not have had a systematic influence on our dependent variable. The pictures were selected from the Chicago Face Database^ 53^ (images nr. CFD-WM-029-023-N and nr. CFD-WM-033-025-N). They were matched with respect to gender, age, ethnicity, and perceived attractiveness. The assignment of each image to a given speaker as well as the distribution of standard versus dialectal morphosyntax to a speaker for a given utterance was counterbalanced across lists.

### Procedure

For the pupillometry experiment, participants were seated in an adjustable chair in a dimly lit room. Although participants’ eyes were tracked in head-free mode, all test subjects placed their head on a chinrest to provide stability and ensure a constant distance between the screen and the participants’ eyes of around 60 cm.

Participants then followed written instructions on the screen (screen without frame = 54.2 cm x 30.5 cm] to calibrate the eye-tracker and to complete the experiment. During the experiment, the dialogue was presented via headphones at a comfortable listening level, and the pupil size of the participant’s dominant eye was recorded at 1000 Hz using an *EyeLink* system^54,55^. Each trial consisted of an image of a male speaker and one utterance. To make sure that participants paid close attention to the materials, they were asked to judge the grammaticality of each utterance, similar to the original study.

For each trial, the picture was displayed before utterance onset for at least 1000 ms. If the participant looked at the picture for at least 400 ms before utterance onset, the presentation of the utterance was initiated. If the participant did not look at the picture, and consequently, the eyetracker recorded trackloss, the picture was continued to be displayed until there was an uninterrupted period of 400 ms during which the participant looked at the picture. This ensured that the pupil size before audio onset could be used as baseline for the pupil size analysis. Participants were instructed to continue to look at the picture while they listened to the utterance and make their judgment as quickly and accurately as possible as soon as the utterance had ended, by pressing one of two designated buttons on a button box placed in front of them. 2000 ms after the utterance offset, a frame appeared around the picture to visually signal that the time for the acceptability judgment had elapsed. Participants could briefly close their eyes and relax while the frame was displayed. After 1000 ms, the frame disappeared. The next trial started with a display of either the same or a different picture, depending on whether there was a speaker switch or not. The experiment included two evenly distributed breaks during which participants could rest their eyes.

### Data preprocessing and statistical analysis

Pupil data was first extracted and downsampled to 500 Hz via SR Research Data Viewer (version 4.3.210)^56^ and later preprocessed and analyzed in R (version 4.5.1)^57^. Pupil size data was preprocessed with the aid of the *gazer* package^58^, applying the following steps: First, all practice trials were removed. Then, we corrected pupillary data for blinks by applying a 5-point moving average smooth and linear interpolation in an extended time window of 200 ms on either side of the blink. In a next step, subtractive baseline correction (i.e., pupil size – baseline) was performed. To make sure that the pupil baseline was not impacted by any language processing mechanisms, we chose a time window of 200 ms before the onset of the utterance as baseline duration during which the participants did not listen to any auditory input. Subsequently, artifact rejection was performed. We excluded trials and participants with more than 20% of missing data points and, following recommendations by Mathôt et al.^59^, we removed spurious pupil values from the data set. To remove further rapid pupil size disturbances, the median absolute deviation metric was applied. During the artifact rejection procedure, five participants and a total of 5.02% of the remaining trials were removed from the dataset. Finally, we aligned the event time data such that 0 corresponded to utterance offset, and additionally downsampled the data to a rate of 50 Hz. The window of analysis for pupillometry data extended from utterance offset to the onset of the frame around the picture prompting participants to give their judgment. This period encompassed the peak of pupil dilation and was chosen in keeping with previous studies that also inserted a silent period for pupil measurements between utterance offset and task onset^3^. Note that as for event time alignment, our data were normalized with respect to utterance offset, not onset, because our stimuli varied in length, leading to variable distances between the onset of the utterance and the start of the experimental task, while the distance between utterance offset and task completion was constant. Before analyzing the pupillometry data, we excluded subjects with excessive trial loss, that is > 2.5 *SD* above mean trial loss per at least one of the conditions (*n* = 4), leaving 1,977,058 data points of a total of 76 participants for analysis. All analyses were performed on proportional changes in pupil size.

First, we implemented growth curve analysis (GCA)^60^ to examine pupil size changes over time. GCA allows for the inspection of the non-linear time course of pupil dilations as well as constrictions and provides a finely-grained insight into the cognitive processes involved during auditory sentence comprehension. In addition to visual world eye-tracking studies (see^61,62)^, GCAs are also increasingly used for the assessment of pupillometry data, as task-evoked pupil response curves can be well modelled using polynomial functions (see^3,15)^. More convex pupil response trajectories reflect greater processing demands, as they indicate a more pronounced and temporally compressed pupillary response to increased cognitive load, and are therefore interpreted here as indicating higher effort during specific conditions. For this purpose, we analyzed changes in pupil size data employing linear mixed-effects regressions with the aid of *lme4*^63^ and *lmertest*^64^ in R. Switch, Speaker, and their interaction were entered as fixed effects into the model, and both two-level factors were sum-coded (Switch: NoSwitch = 1, Switch = −1; Speaker: Dialect = 1; Standard German = −1). To determine the change of pupillary responses over time more precisely, first (linear) and second (quadratic) order polynomials and their interactions with Switch and Speaker were added to the model. Additionally, we included the main effect of Trial (scaled and centered) as a covariate to account for general pupil size changes in the course of the experiment. Given that visual inspection of the pupil data showed only one bend in the pupil curve averaged across all participants and trials, we disregarded polynomials beyond the second order (see^62,^^65^, for a similar procedure). The random effect structure included random intercepts for participants and items as well as random slopes for Switch, Speaker, and their interaction, as well as a random slope for Trial for participants. To examine whether switching costs and asymmetries changed over the course of the dialogue, a GCA with a four-way interaction of Trial × Switch × Speaker × Polynomial was considered. However, simultaneously modelling within-trial pupil dynamics and across-trial changes in a single model is conceptually complex, as the two temporal scales reflect qualitatively different processes. Such higher-order interactions substantially increased model complexity and did not converge reliably. They were therefore not pursued further. To inspect adaptation processes across the entire experiment, we computed an additional linear mixed model with pupil size as the dependent variable, which included the three-way interaction of Speaker x Switch x and Trial as predictor. Both two-level factors were sum-coded (see above), and Trial was scaled and centered. The model included by-subject random intercepts with random slopes for the three-way interaction of Switch x Speaker x Trial, as well as by-item random intercepts.

To identify distinct listener profiles for switching patterns, we performed k-means cluster analysis with the aid of the *stats* package^57^. This method clusters participants into *k* groups, aiming to maximize similarity among subjects within the same cluster based on the criteria entered in the analysis^66^. For this purpose, we calculated mean difference scores for switching cost and switching asymmetry per participant and condition for pupil size averaged over the time window ranging from 0 to 2000 ms after utterance offset. The switch cost (switch – no switch) reflects the ease of switching between speakers. The switch asymmetry (standard-to-dialect switch – dialect-to-standard switch) indexes the additional cognitive effort required during across-speaker switches (i.e., how much more effortful it is to switch into dialect than to switch into standard). In addition to these two factors, we also included individual differences in age, German proficiency, Standard German input, dialect input, input from other languages, social network size, and the Big Five personality traits extraversion, neuroticism, openness, conscientiousness, and agreeableness, as well as the speaker comprehensibility and pleasantness difference scores in the analysis. All factors were scaled and centered. To select the optimal number of clusters (*k*), we examined the within-cluster sum of squares across up to 10 clusters, suggesting a 2-cluster solution for further analysis. To determine whether the two clusters differed across the above-mentioned variables, we conducted independent *t*-tests, with *p*-values adjusted using Holm-Bonferroni correction to control for multiple comparisons.

## Data Availability

The materials, data, and data analysis scripts are available upon request (hanulikova@idf.uni-heidelberg.de).
